# Adipose Tissue Dysfunction Induced by High-Fat Diet Consumption Is Associated with Higher Otoacoustic Emissions Threshold in Mice C57BL/6

**DOI:** 10.3390/nu17111786

**Published:** 2025-05-24

**Authors:** Gonzalo Terreros, Felipe Munoz, Matías Magdalena, Manuel Soto-Donoso, Nairo Torres, Amanda D’Espessailles

**Affiliations:** 1 Laboratorio de Neurociencia Sensorial, Perceptual y Cognitiva, Instituto de Ciencias de la Salud, Universidad de O’Higgins, Rancagua 2820000, Chile; gonzalo.terreros@uoh.cl (G.T.); felipe.munoza@uoh.cl (F.M.); manuel.soto@pregrado.uoh.cl (M.S.-D.); 2 Escuela de Salud, Universidad de O’Higgins, Rancagua 2820000, Chile; 3 Doctorado en Ciencias de la Bioingeniería, Universidad de O’Higgins, Rancagua 2820000, Chile

**Keywords:** adipose tissue, hearing loss, obesity, adipose tissue dysfunction

## Abstract

**Background/Objectives**: Obesity is a risk factor for several diseases; however, less has been researched about how diet-induced obesity may affect the auditory system. In this sense, the purpose of this study was to evaluate the effect of diet-induced obesity on the functionality and integrity of the outer hair cells, a key component of the organ of Corti, inside the cochlea. Furthermore, we hypothesized that adipose tissue (AT) status is associated with impaired outer hair cell auditory amplification in young C57BL/6 mice, contributing to increased vulnerability to hearing damage. **Methods**: Weaning male C57BL/6J mice (7 weeks old) weighing 22–23 g were divided into two diet groups: (i) a control diet or (ii) a high-fat diet (HFD) for 12 or 16 weeks. Metabolic parameters (body and AT weight, glucose tolerance test), AT dysfunction markers (AT remodeling, adipocyte size, crown-like structures), and outer hair cell function (distortion products otoacoustic emissions (DPOAEs) threshold and amplitudes) and integrity (hair cells cell count) were evaluated. **Results**: We observed that mice fed an HFD for 16 weeks showed a higher DPOAE threshold against stimuli at 16 KHz and a lower count of outer hair cells in the medial section of the cochlea. These results demonstrate a correlation between body and AT weight specifically at 16 weeks of treatment, the time point at which we observed a marked AT dysfunction. **Conclusions**: Taken together, our results suggest that obese mice with AT dysfunction have an altered auditory efferent system, characterized by a higher DPOAE threshold and a lower outer hair cell count in the medial section, which may impact signal transduction.

## 1. Introduction

Obesity, an excess in body fat accumulation in the adipose tissue (AT), is a growing global health concern [1]. Interestingly, a growing body of epidemiological evidence suggests that obesity, particularly central obesity (characterized by fat accumulation in the abdominal region), could also affect the auditory system [2,3,4]. This link positions obesity and its associated comorbidities as potential risk factors for the development of hearing loss in humans [5]. While obesity’s impact on hearing has been studied regarding general auditory function and sensory hair cells, its potential effects on outer hair cell function and integrity remain largely unexplored.

Hearing loss refers to the decrease or inability to perceive sounds in one or both ears, requiring the sound to have a higher sound pressure to be heard [6]. It is a dominant handicap in modern society, affecting 466 million people worldwide, 34 million of them being children. Projections indicate a substantial increase, with an estimated 2.45 billion people experiencing hearing loss by the year 2050 [7]. Hearing loss represents a modifiable risk factor that has been consistently associated with impairments in cognitive skills and an increased risk of dementia in the aging population [8,9].

Signal transduction in the auditory system occurs within the organ of Corti, a specialized structure in the cochlea. Inside the cochlea, the sensory cells, named hair cells, are the sensory cells that transform sound waves into electrical impulses. This physiological process allows the transmission of these signals from the auditory nerve to the brain, where they are perceived and interpreted as sounds [10]. In this context, damage to the hair cells within the organ of Corti, the auditory nerve fibers connecting them to the brainstem, or the synaptic connections between these structures can lead to irreversible hearing loss. This damage disrupts the critical process of transducing sound signals into neural impulses, resulting in difficulties with detecting and interpreting auditory stimuli [11]. In this sense, hearing loss has a complex and diverse etiology. Several factors affect the auditory system and impair it. Sensorineural hearing loss is the most prevalent type of hearing loss and is associated with the loss of hair cells in the organ of Corti, and dysfunction and degeneration of stria vascularis and spiral ganglion cells [12]. Two of the pathophysiological processes related to hair cell death and sensorineural hearing loss are oxidative stress and inflammation, which is a pathophysiological mechanism that underlies drugs, noise, or aging-induced hearing loss (all subtypes of sensorineural hearing loss) [13]. Considering that obesity can be associated with a chronic and low-grade inflammatory process, it is interesting to study how inflammation induced by expanding AT could impact the auditory system in this context.

Similarly, obesity is also a significant global health concern, with projections estimating that over half of all adults worldwide will be overweight or obese [14]. Obesity has reached pandemic proportions globally, affecting about 13% of the adult population. This is especially troublesome considering that the prevalence of childhood obesity has also risen dramatically, with over 340 million children and adolescents aged 5–19 now classified as overweight or obese [14,15]. Thus, studying the specific relationship between obesity, overweight, and hearing loss is crucial for the present and future global outlook. In this sense, it has been widely reported that AT expansion, especially in the visceral AT depot, triggers a proinflammatory response that leads to AT dysfunction. AT dysfunction is defined as an imbalance in the adipocyte secretory profile, increasing the secretion of proinflammatory over anti-inflammatory adipokines, and thus, altering the homeostasis of the whole organism [16]. For example, dysfunctional AT shows increased secretion of proinflammatory cytokines such as IL-6, IL-1β, and tumor necrosis factor (TNF)-α, and lower secretion of anti-inflammatory adipokines such as adiponectin. In this context, a recent work by Chan et al. [17] associated inflammation in the cochlea with high-fat diet (HFD) consumption. After 10 weeks of HFD, they observed an increase in the immuno-expression of proinflammatory biomarkers such as intracellular adhesion molecule 1, IL-6 receptor-α, and toll-like-receptor 2 in the cochlea. Moreover, this inflammatory state was associated with the presence of macrophages in the lateral wall of the cochlea. Furthermore, another study observed that consumption of an HFD induces the expression of TNF-α and nuclear factor (NF)-κB in the cochlea of mice [18]. Taken together, these findings show an association between HFD and cochlear inflammation, suggesting a role for AT dysfunction in cochlear damage and hearing impairment.

Despite its importance, the specific impact of AT expansion and AT dysfunction on hearing remains largely unknown. Understanding how adipose tissue dysfunction relates to hearing impairment, specifically how it may affect outer hair cell function and integrity, could provide essential insights into obesity-related hearing disorders and potentially reveal new therapeutic targets. Therefore, this study aims to evaluate the effect of diet-induced obesity and consequent adipose tissue dysfunction on the functionality and integrity of outer hair cells in young C57BL/6 mice.

## 2. Materials and Methods

### 2.1. Ethics Statement

Experimental animal protocols and animal procedures complied with the Guide for the Care and Use of Laboratory Animals (National Academy of Sciences, NIH Publication 6-23, revised 1985) and were approved by the Institutional Committee for the Care and Use of Animals, Universidad de O’Higgins (CICUA 012-2024 UOH).

### 2.2. Animal Preparation

Weaning male C57BL/6J mice (7 weeks old, n = 40) weighing 22–23 g were obtained from the Animal Facility at the Institute of Public Health, Chile. The animals were related but not all of them were from the same litter. Room temperature was constant at 21 °C, and the light was maintained on a 12:12-h light–dark cycle. After one week, the mice were randomly divided into two diet groups (n = 20 for each group)—(i) control diet (CD, Research Diets Inc., D12450B, New Brunswick, NJ, USA) containing 10% fat, 20% protein, and 70% carbohydrate (expressed as % total calories), with a caloric value of 3.85 kcal/g, or (ii) HFD (Research Diets Inc., D12492, USA) containing 60% fat, 20% protein, and 20% carbohydrate, with a caloric value of 5.24 kcal/g—for 12 or 16 weeks, resulting in groups of 10 animals per group. Weekly controls of body weight and diet intake were performed throughout the period. At the end of treatment, animals were fasted (6–8 h) and then anesthetized with ketamine (100 mg/kg) and xylazine (10 mg/kg ip).

### 2.3. Glucose Tolerance Test and Biochemical Determinations

A glucose tolerance test was performed on the animals at 12 and 16 weeks of treatment (n = 5 for each group), 2 days before euthanasia. Mice were subjected to a 6-h fast and blood glucose (mg/dL) was measured at time zero using a glucometer (One Touch Glucometer, Johnson and Johnson, New Brunswick, NJ, USA), following the manufacturer’s instructions. Glucose (1.5 mg/g of body weight) was injected ip and blood glucose levels were measured at 15-, 30-, 90- and 120-min post glucose intake. The data obtained were analyzed and plotted, and the area under the curve was calculated.

### 2.4. Evocated Otoacoustic Emissions

Distortion products otoacoustic emissions (DPOAEs) were measured (n = 5 for each group) under anesthesia (100 mg/kg ketamine and 2.5 mg/kg xylazine), maintaining a constant temperature (37 °C) in a double-walled sound-proofed room. The 2f1-f2 DPOAE was measured for f2 frequencies of 8, 16, and 20 KHz in the left ear for all animals. DPOAE was measured using an ER10B+ high-frequency microphone (Etymotic Research, Elk Grove Village, IL, USA) and two MF-1 transducers (Tucker-Davis Technology, Alachua, FL, USA). Two primary tones (L1-L2 = 5 dB SPL), were presented through calibrated headphones with a primary frequency ratio (f2/f1) of 1.25, starting at L1 = 65 dB SPL. DPOAE amplitudes, were obtained at L1 of 60, 50, 40, 30, and 20-dB SPL. Data were computed using a custom-made MATLAB v24.1 program and a USB-6346 data acquisition device from National Instruments Corporation (Austin, TX, USA). Finally, the DPOAE threshold was defined as the lower dB where a DPOAE was observed. The results for each animal were averaged for each test frequency and then used to calculate group mean data. All the analyses were performed independently in a blind fashion by two of the authors to avoid bias.

### 2.5. Tissue Samples and Histological Analysis

Visceral adipose tissue (epididymal area) was weighed and fixed in phosphate-buffered formalin, embedded in paraffin, sectioned, and stained with hematoxylin–eosin (HE). The slides were stained with HE and analyzed by optical microscopy (Olympus CX31, Olympus, Tokyo, Japan) for morphology analysis in a blind fashion. Adipocyte area (size in μm^2^), % of extracellular matrix, and number of crown structures were assessed with ImageJ 1.46r (National Institutes of Health, Bethesda, MD, USA) software in HE-stained slides in at least five adjacent fields. Cochlear tissue was extracted and either frozen in liquid nitrogen for ARN extraction or fixed in paraformaldehyde 4% for 24 h for immunostaining.

### 2.6. Cochlear Immunofluorescence

The cochlea was extracted, fixed with 4% paraformaldehyde in phosphate buffer overnight, and decalcified with 100 mM EDTA in PBS for 5 days at 4 °C. Decalcified cochleae were then dissected into apical, medial, and apical sections [19]. The cochlear sections were blocked and permeabilized with normal goat serum (Jackson Immuno Research Labs, West Grove, PA, USA) for 1 h, washed, and then incubated overnight with primary antibody to Myo7a (25-6790 Proteus Biosciences, Ramona, CA, USA), a specific marker for hair cells. Negative controls were prepared in parallel under identical conditions but incubated without primary antibodies. After incubation, sections were washed three times in PBS and incubated with a corresponding secondary antibody (Alexa Fluor 594, IgG, ThermoFisher, Waltham, MA, USA) diluted in PBS for 2 h and then marked for 30 min with Phalloidin Alexa Fluor TM A12379 (Invitrogen, Waltham, MA, USA). Images of immunolabeled sections were obtained by epifluorescence microscopy using a ZEISS Axio Observer 7 microscope and ZEISS Apotome 3 for optical sectioning (Zeiss Group, Jena, Germany). The number of inner and outer hair cells in the organ of Corti was assessed using ImageJ 1.46r (National Institutes of Health, EE.UU.) blindly by one of the authors and analyzed and plotted by another to avoid bias. The data were presented as the number of cells per 100 µm.

### 2.7. Statistical Analysis

Statistical analysis was carried out using GraphPad Prism v 10.0.2. All data are expressed as mean ± standard error (SEM). Data normality was tested for all results. Weight increase and glucose tolerance were analyzed by a multiple Student’s *t*-test; general parameters, glucose AUC, adipose size and number of crown-like structures, DPOAE threshold and amplitude, and hair cell count, were analyzed by two-way ANOVA followed by Tukey’s. Pearson correlation analysis was used to evaluate the relations between variables. The statistical significance level was defined at *p* < 0.05.

## 3. Results

### 3.1. HFD Induces Weight Gain and Adipose Tissue Dysfunction

**Figure 1:**
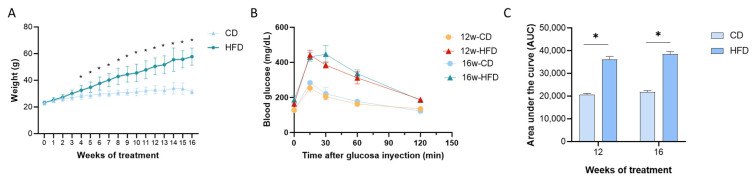
HFD diet increases body weight gain and decreases glucose tolerance at 12 and 16 weeks of treatment. (**A**) Animals were fed a control diet (CD, low-fat diet, 20 kcal% fat content diet) or high-fat diet (HFD, 60 kcal% fat content), and body weight was evaluated weekly. (**B**) Glucose tolerance test and (**C**) related area under the curve (AUC) were measured at 12 and 16 weeks of treatment (n = 5 for each group). Values are expressed as mean ± SEM. * for statistically significant differences between groups at each time (multiple *t*-test for (**A**,**B**); two-way ANOVA and Tukey’s post-test for (**C**), *p* < 0.05).

Animals were fed CD or HFD for 12 or 16 weeks to evaluate the effect of those diets on hearing system function and integrity. At the start of the treatment, there was no difference in the initial body weight among all the animals (CD: 23.2 ± 1.48; HFD: 22.8 ± 2.2 g). As expected, 12 and 16 weeks of HFD significantly increased body weight (diet: F (1, 29) = 96) and visceral adipose tissue (VAT) weight (diet: F (1, 20) = 79; weeks: F (1, 20) = 6.2; and interaction: F (1, 20) = 11) (Table 1), without changes in liver weight, compared to the CD-fed groups. Moreover, HFD increased body weight from the fourth week compared to animal-fed CD (*p* < 0.05, *t*-test) (Figure 1A). Additionally, we measured blood glucose levels and glucose tolerance tests in mice at the start of weeks 12 and 16 of treatment, 2 days before euthanasia. HFD decreased glucose tolerance (Figure 1B), increasing the area under the curve of glucose in 12 and 16 weeks (diet: F (1, 19) = 182.4, *p* < 0.0001) compared to CD (Figure 1C), indicating that length of treatment does not affect glucose levels at this time (F (1, 19) = 2.158, *p* = 0.1582).

**Figure 2:**
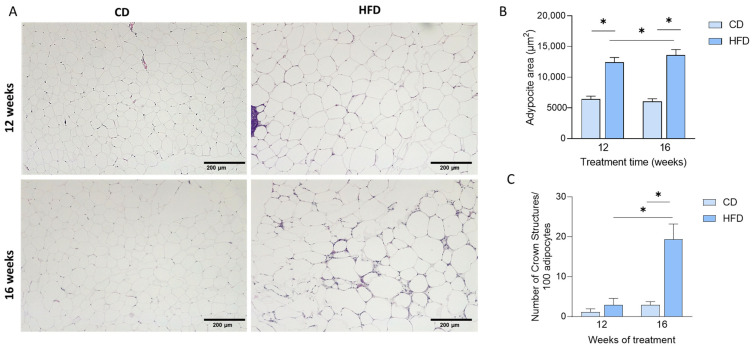
HFD diet induces adipose tissue dysfunction markers at 16 weeks of treatment. (**A**) Representative image of adipose tissue sections stained with HE of animals fed a control diet (CD, low-fat diet, 20 kcal% fat content diet) or high-fat diet (HFD, 60 kcal% fat content) for 12 and 16 weeks. (**B**) HFD increased the adipocyte area independent of treatment time while only increasing crown structure numbers after 16 weeks of treatment (**C**). Values are expressed as mean ± SEM; n = 5 for each group. * for statistically significant differences between groups at each time (two-way ANOVA and Tukey’s post-test, *p* < 0.05).

Moreover, when studying histological changes in AT sections (Figure 2A), we observed that HFD at 12 and 16 weeks increased adipocyte area (diet: F (1, 11) = 72; *p* < 0.001; Figure 2B), while the time of treatment had no effect. Interestingly, analyzing the number of crown structures present in the histological sections, we observed that animals fed an HFD for 16 weeks but not 12 weeks (Figure 2C) had a significantly higher number of these structures (interaction: F (1, 17) = 8.6, *p* = 0.00094; weeks: F (1, 17) = 9.4, *p* = 0.0069; diet: F (1, 17) = 13, *p* = 0.023). In line with these results, VAT extracellular matrix content (% ECM area) also increased in mice fed 16 weeks with HFD (diet: F (1, 67) = 10; weeks: F (1, 67) = 265; and interaction: F (1, 67) = 7.8), compared to CD and 12 weeks of treatment (Table 1).

### 3.2. HFD Treatment Increased DPOAE Threshold at 16 KHz After 16 Weeks of Treatment

**Figure 3:**
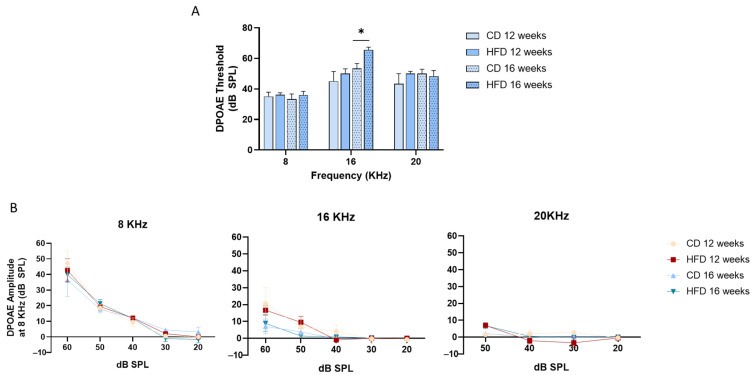
HFD treatment increased the DPOAE threshold after 16 weeks of treatment at 16 KHz. Distortion product otoacoustic emissions (DPOAEs), an indirect measure of outer hair cell (OHC) function, were evaluated at 12 and 16 weeks of treatment at an F = 2 frequency of 8, 16, and 20 KHz. DPOAE threshold (**A**) at 16 KHz was increased by HFD at 16 but not 12 weeks of treatment. (**B**) DPOAE amplitudes at 8, 16, and 20 KHz were not affected by diet or treatment time. Values are expressed as mean ± SEM; n = 5 for each group. * for statistically significant differences between groups at each time (two-way ANOVA and Tukey’s post-test, *p* < 0.05).

Distortion product otoacoustic emission (DPOAE) thresholds were recorded from the ear canal following tone stimulation (f1 and f2) to assess outer hair cell electromotility. Considering the essential role of OHC function in amplifying the vibrations of the basilar membrane in the cochlea, and increasing the sensitivity and selectivity of inner hair cells, we investigated whether an HFD could alter DPOAE thresholds after 12 and 16 weeks of treatment. Anesthetized mice were evaluated and DPOAE thresholds were recorded at 8, 16, and 20 kHz. As shown in Figure 3A, DPOAE thresholds were significantly elevated at 16 kHz in 16-week-treated mice (diet: F (1, 15) = 8.5, *p* = 0.0106) compared to CD-fed mice. Conversely, DPOAE thresholds remained unchanged at 8 and 20 kHz, suggesting that HFD affects outer hair cell amplification capacity specifically in mice’s medial region of the cochlea. Furthermore, HFD consumption did not alter DPOAE thresholds at any of the tested frequencies (8, 16, and 20 kHz) in mice after 12 weeks. These results suggest that the impact of HFD on outer hair cell function, as measured by DPOAE thresholds, may require a longer duration of exposure to manifest, at least at the frequencies examined in this study.

In addition to the DPOAE threshold, we measured distortion amplitudes at 8, 16, and 20 KHz at stimulus levels of 60 (except 20 KHz), 50, 40, 30, and 20 dB SPL (Figure 3B). Our analysis revealed that neither diet nor treatment duration significantly affected the DPOAE amplitude.

### 3.3. HFD Consumption for 16 Weeks Impairs Outer Hair Cells in the Medial Cochlea

**Figure 4:**
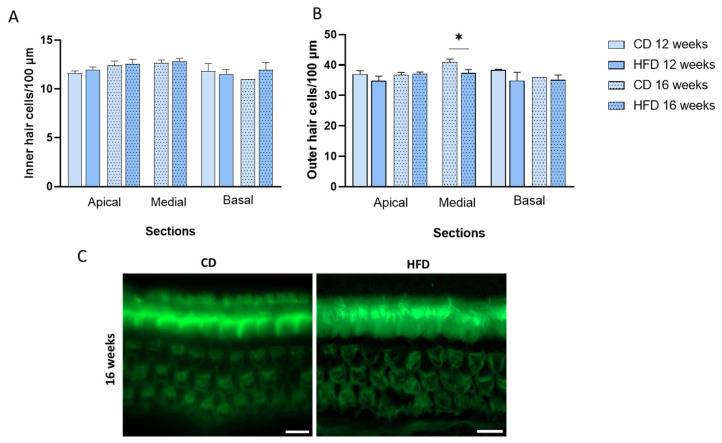
HFD consumption for 16 weeks impairs outer hair cells in the medial cochlea. Whole-mount preparations of the auditory epithelium were dissected into apical, medial, and basal sections, stained for Phalloidin, and photographed using epifluorescence. (**A**) Number of inner and (**B**) outer hair cells per 100 μm. (**C**) Representative image of inner and outer hair cells of the organ of Corti of a medial section from animals fed a control diet (CD) or high-fat diet (HFD) for 16 weeks. Scale bar = 10 μm. Values are expressed as mean ± SEM; n = 3–5 for each group. * for statistically significant differences between groups at each time (two-way ANOVA and Tukey’s post-test, *p* < 0.05).

After DPOAE measurement, we studied the cellular structure of the inner and outer hair cells in the organ of Corti, represented by Figure 4B. Both inner and outer hair cells were marked with Myo7a and Phalloidin staining in the whole-mount section of the cochlea (apical, medial, and basal). We observed that inner hair cells were not affected by diet selection or treatment time. However, animals fed with HFD for 16 weeks presented fewer outer hair cells than CD-fed animals (*p* = 0.0214) in the medial section of the organ of Corti but not in the apical or basal sections (Figure 4B).

### 3.4. DPOAE Threshold and DPOAE Amplitudes Differentially Correlate with Body Weight and Adipose Tissue (AT) Weight Dependent on Length of Treatment

**Figure 5:**
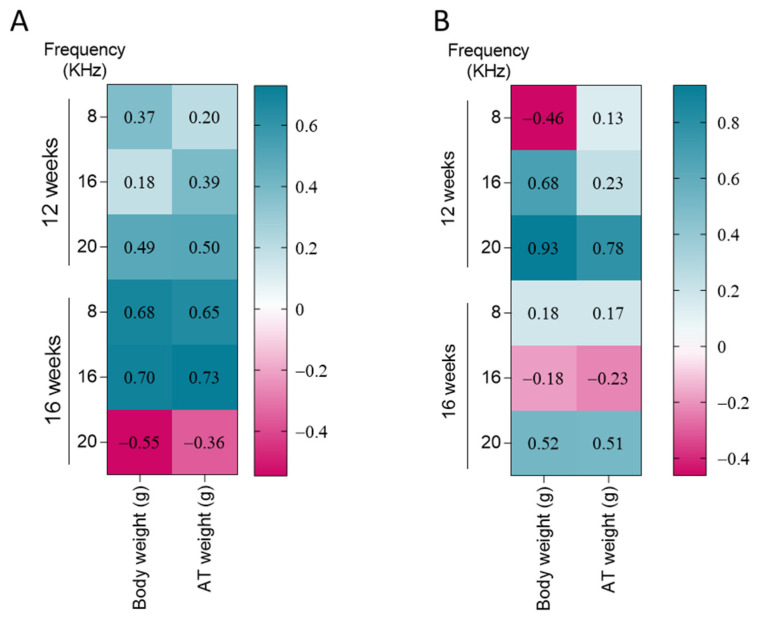
DPOAE threshold and DPOAE amplitudes differentially correlate with body weight and adipose tissue (AT) weight dependent on the length of treatment. Hot maps of Pearson correlation between DPOAE threshold (**A**) and DPOAE amplitudes (**B**) and body and AT weight at 12 and 16 weeks of treatment, for all the frequencies measured. Pearson’s factor r is depicted inside the hot maps.

To associate metabolic status with hearing functionality, we prepared a Pearson correlation matrix to assess the association between DPOAE threshold and amplitude (as a measure of outer hair cells electromobility) with body weight and AT weight (Figure 5). A higher DPOAE threshold indicates a lower functionality of outer hair cells, while a higher amplitude indicates a stronger function of these cells [20]. Interestingly, we observed that body and AT weight positively correlate with the DPOAE threshold at 8 (body weight, *p* = 0.015; AT weight, *p* = 0.02) and 16 KHz (body weight, *p* = 0.012; AT weight, *p* = 0.08), only at 16 weeks of treatment, but not at 12. Moreover, at a frequency of 20 KHz, there was no significant correlation between the DPOAE threshold and the metabolic parameters assessed (Figure 5A). On the other hand, when we evaluated the association between DPOAE amplitudes with body and AT weight we observed a significant positive correlation between DPOAE amplitudes and body and AT weight at 16 (body weight, *p* = 0.045) and 20 KHz (body weight, *p* = 0.001; AT weight, *p* = 0.019) in the animals fed for 12 weeks (Figure 5B). At 16 weeks of treatment, the only significant correlation was found at 20 KHz (body weight, *p* = 0.013; AT weight, *p* = 0.011).

## 4. Discussion

**Figure 6:**
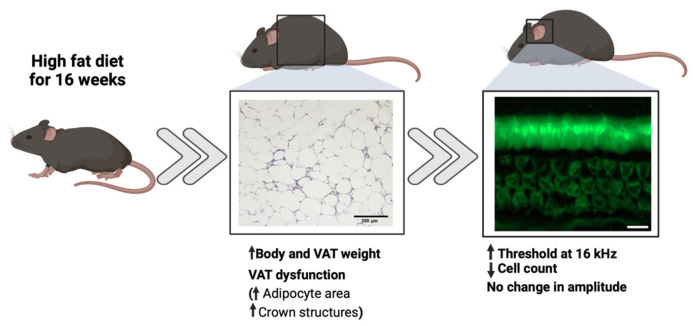
Graphical summary of the main findings after 16 weeks of high-fat diet (HFD) in mice. HFD induced increased body weight, visceral adipose tissue (VAT) weight, and signs of VAT dysfunction such as adipocyte hypertrophy and crown-like structures (middle panel, H&E staining; scale bar = 20 µm). In the cochlea (right panel), an increased DPOAE threshold at 16 kHz and a decreased hair cell count were observed, with no significant changes in DPOAE amplitude.

Mice fed a high-fat diet (HFD) is one of the most studied models of obesity-induced metabolic alterations [21]; however, less has been researched about how diet-induced obesity may affect the auditory system. In this sense, we aimed to evaluate the effect of diet-induced obesity on the functionality and integrity of the outer hair cells, a key component of the organ of Corti. Furthermore, we hypothesized that adipose tissue (AT) status is associated with impaired outer hair cell auditory amplification in young C57BL/6 mice, contributing to increased vulnerability to hearing damage. We observed that mice fed an HFD for 16 weeks showed a higher DPOAE threshold against stimuli at 16 KHz and a lower count of outer hair cells in the medial section of the cochlea (Figure 6). These results demonstrate a correlation between body and AT weight specifically at 16 weeks of treatment, the time point at which we observed a marked AT dysfunction.

Epidemiological studies have observed a strong association between obesity and sensorineural hearing loss (SNHL) [5], suggesting that overweight and obesity may be a risk factor for hearing loss in humans. However, most of these studies evaluated obesity using BMI, associating hearing loss with general obesity and making it difficult to assess the AT contribution to hearing impairment. In this sense, Hwang et al. [3] observed an association between central obesity and the severity of age-related hearing impairment (in low frequencies for men under 55 years and in high frequencies for women over 55 years). Moreover, they described a positive correlation between waist circumference (used to measure visceral AT) and hearing damage, independent of age. In the same line, Kim et al. [22] concluded that visceral AT content (evaluated by computational tomography) positively correlates with HL in women (increased low and high-frequency hearing thresholds), independent of age. These data suggested that fat localization may be a critical issue in the obesity and HL relationship.

In diet-induced obesity and metabolic dysfunction, visceral AT expansion has been strongly associated with initiating chronic and low-grade metabolic inflammation [16]. This scenario triggers an imbalance in adipokine production, promoting the secretion of proinflammatory over antiinflammatory adipokines [23], known as AT dysfunction [24]. AT dysfunction, especially visceral AT, is characterized by enhanced macrophage infiltration, inflammation, and remodeling of adipose tissue [25]. Macrophages and other leukocytes infiltrate the AT and surround the hypertrophic adipocytes, forming crown-like structures [26]. In this respect, we observed that animals fed an HFD for 16 weeks (but not 12 weeks) developed AT dysfunction. This state was characterized by a significant increase in visceral AT weight, AT remodeling (increased extracellular matrix content), adipocyte hypertrophy, and a higher number of crown-like structures in the AT, as has been described before [25,26,27].

AT dysfunction affects the whole organism triggering a systemic pro-oxidant and proinflammatory reaction [16]. Of note, AT dysfunction has been proposed to determine lipotoxicity, trigger metabolic syndrome (that includes insulin resistance) [28], and signal hepatic steatosis progression [29] in mice. However, its possible role in hearing impairment is unclear.

Several recent studies have evaluated the effect of HFD on hearing functionality in animal models, yielding conflicting results. We observed that C57BL/6J mice fed an HFD for 16 weeks presented a higher DPOAE threshold at 16 KHz and a decrease in outer hair cell number in the medial section compared to control diet (CD)-fed animals. In line with our results, HFD consumption for 12 and 16 weeks deteriorated hearing function in mice strains such as CBA [30] and CD/1 [18] by inducing mitochondrial dysfunction that increases reactive oxygen species (ROS) production and oxidative stress, leading to apoptosis of hair and spiral ganglion cells (cells that relay acoustic information from sensory hair cells to central auditory circuits) in the cochlea. Moreover, C57BL/6J mice fed a high-fat and high-fructose diet for 12 weeks had significant hearing impairment, as well as increased levels of ROS and hair cell death due to reduced levels of pAKT and superoxide dismutase 2 (SOD2) in the cochlea.

In contrast to this evidence, Fujita et al., 2015 [31] observed that consumption of an HFD for 3 and 5 months increased the auditory brainstem response (ABR) threshold (a measurement of hearing function) at 4 and 32 KHz in C57BL/6J mice. Moreover, after 12 months, HFD consumption suppressed spiral ganglion cell loss and elevation of the ABR threshold, delaying age-related hearing loss in this model. However, they did not evaluate the function or integrity of outer hair cells. The authors proposed two mechanisms that may explain HFD protection against age-related hearing loss: (i) the antioxidant effect of high levels of vitamin E found in the HFD, and (ii) intrinsic resistance of C57BL/6J mice to glucose intolerance, which would minimize the damage from high blood glucose. However, they also observed that HFD consumption at the same time worsened hearing function in CBA/N-slc mice, which suggests that the effect of HFD may be related to the intrinsic characteristics of the C57BL/6J strain more than the diet composition. Similarly, Zhang et al., 2023 [32], concluded that an HFD had a rescuing effect on age-related hearing loss in aged C57BL/6J mice, which was associated with a better conservation of outer hair cell sizes and prestin levels (a transmembrane motor protein that facilitates outer hair cell electromobility) at 12 months. They observed that HFD prevented age-induced damage in ABR and DPOAE thresholds and amplitudes from 8 months of treatment. In addition, they also studied the effect of the diet on CBA/CaJ mice and observed similar results to those of Fujita et al. [31], where HFD worsened hearing function in aged mice. The authors observed that C57BL/6J presented a downregulation of arachidonic acid (an inflammatory fatty acid), and a reduced inflammatory response, which may protect the cochlea from age-induced damage.

Interestingly, the C57BL/6J strain presents a genetically progressive sensorineural hearing loss that promotes early onset of age-related hearing loss (at 12 months), compared to other mice strains [33], due to the presence of a specific mutation (Cdh23^753A^) [34]. This sensibilizes cochlear cells to oxidative stress, triggering mitochondrial-dependent apoptosis [33], making C57BL/6J mice’s cochlea more susceptible to oxidative stress associated with aging damage. In this context, obesity triggers low-grade oxidative stress [23], which could induce mitochondrial preconditioning on cochlear tissue [35], thus preventing further oxidative injury by aging in these mice. This observation explains the paradox of HFD protection on hearing damage in C57BL/6J aged mice, considering that diet protection is only described in hearing function [32]. On the other hand, we did not evaluate the effect of aging on hearing function, studying the impact of HFD at 19 and 23 weeks (HFD was given for 12 or 16 weeks to 7-week-old mice), when the animals are considered young in terms of hearing [12].

As discussed, AT dysfunction may have a role in the hearing impairment observed in obesity. AT dysfunction is characterized by altered adipokine secretion, particularly reduced adiponectin, which has protective effects on cochlear structures [30,36]; it also induces chronic low-grade inflammation with increased proinflammatory cytokines that could reach the cochlea [17], and increases oxidative stress due to excessive ROS production that could exceed antioxidant defenses in sensitive tissues such as the cochlea and trigger mitochondrial-dependent apoptosis [37]. In this sense, we observed that DPOAE detriment positively correlates with body and AT weight at 8 and 16 KHz at 16 weeks of treatment. Interestingly, we only observed AT dysfunction markers at 16 weeks, but not at 12 weeks, which could suggest that AT metabolism may have a role in hearing health. Moreover, we also observed that DPOAE amplitude positively correlates with auditory efferent system function at 12 weeks, but not at 16 weeks of treatment. Taking together, our results suggest that obese mice with AT dysfunction have an altered auditory efferent system, characterized by a higher DPOAE threshold and lower outer hair cell count in the medial section, which may impact signal transduction.

Notably, we observed changes at 16 KHz but not at higher frequencies. In this sense, the literature shows that hearing damage starts in the basal section of the cochlea and usually correlates with increased thresholds in ABR and lower DPOAE amplitudes at high frequencies [12]. However, in obesity-induced hearing damage, the data are more controversial, with findings that observed damage in the high-frequency regions [18,32,37], and others in low and high frequencies [30,31], without a clear pattern among the different measurements. These data support the idea that in diet-associated hearing impairment, damage progression may not always be linear from basal to apical regions. One explanation for these observations is the effect of lipids on outer hair cell electromobility. It is known that HFD consumption increases the proportion of saturated fatty acids and cholesterol in cell membranes [38]. In this sense, studies show that elevated cholesterol levels can reduce prestin (a motor protein essential for electromotility in outer hair cells) movement and function [39], altering the motility of outer hair cells. Considering that prestin is more expressed in the basal section of the cochlea compared to the medial section [40], it may be possible that lipid membrane alteration affects outer hair cell function in the medial section, which correlates to the higher DPOAE threshold observed at 16 KHz.

Some limitations of this study are the small sample size and the exclusion of female subjects. Increasing the sample size could have made our results more robust. Including female subjects would have improved our conclusions, considering that research including females is limited. However, we only included males considering that it has been described that female mice are protected against obesity-induced hearing damage [30] and that being male is a risk factor for hearing loss [41].

## 5. Conclusions

Our results suggest that obesity—characterized as an increased body and visceral AT weight—may affect hearing function at an early age in mice. In the medial cochlear section, we observed that consumption of an HFD for 16 weeks was detrimental to outer hair cell functionality (evaluated as DPOAE threshold) and integrity (evaluated as number of cells). Moreover, this effect on outer hair cells was correlated to body and adipose tissue weight only in mice with adipose tissue dysfunction. These data contribute to the growing understanding of metabolic alterations and hearing impairment and help us to understand the role of adipose tissue in hearing.

## Figures and Tables

**Table 1:** General parameters.

	12 Weeks		16 Weeks		*p* (2-Way ANOVA)		
	CD	HFD	CD	HFD	Diet	Weeks	Interaction
Final weight (g)	32.1 ± 2.48 ^a^	50.3 ± 5.23 ^b,d^	31.3 ± 1.5 ^a,c^	57.8 ± 6.4 ^d^	<0.0001	0.0776	0.2036
Liver weight (g)	1.42 ± 0.19 ^a^	2.03 ± 0.61 ^b^	1.4 ± 0.056 ^a,b^	2.1 ± 0.17 ^a,b^	0.003	0.98	0.80
Liver/body weight ratio	0.044 ± 0.0043	0.041 ± 0.011	0.044 ± 0.0014	0.036 ± 0.0044	0.1964	0.4809	0.4992
VAT weight (g)	0.96 ± 0.30 ^a^	2.07 ± 0.53 ^b^	0.8 ± 0.2 ^a,c^	3.2 ± 0.45 ^d^	<0.0001	0.0214	0.0036
VAT % ECM area	0.65 ± 0.10 ^a^	0.84 ± 0.19 ^a^	8.02 ± 1.7 ^b^	11.77 ± 3.7 ^c^	0.0068	<0.001	0.0024

Values are expressed as mean ± SEM. CD: control diet (12 weeks, n = 10; 16 weeks, n = 10); HFD: high-fat diet (12 weeks, n = 10; 16 weeks, n = 10). VAT: visceral adipose tissue; ECM: extracellular matrix. Differences between groups were measured by two-way ANOVA and Tukey’s post-test, *p* < 0.05 for statistically significant differences are indicated by different letters.

## Data Availability

Data are available upon request.

## Abbreviations

The following abbreviations are used in this manuscript:

AT	Adipose tissue
CD	Control diet
DPOAE	Distortion product otoacoustic emission
ECM	Extracellular matrix
HFD	High-fat diet
ROS	Reactive oxygen species
SNHL	Sensorineural hearing loss
TNF-α	Tumor necrosis factor-α
VAT	Adipose tissue
